# Antibody-drug conjugates in breast cancer: current resistance mechanisms and future combination strategies

**DOI:** 10.20517/cdr.2025.26

**Published:** 2025-06-17

**Authors:** Ping Xing, Chenghui Yang, Hanwen Hu, Tianyi Qian, Bojian Xie, Jian Huang, Zhen Wang

**Affiliations:** ^1^Department of Breast Surgery, Second Affiliated Hospital, Zhejiang University School of Medicine, Hangzhou 310009, Zhejiang, China.; ^2^Key Laboratory of Tumor Microenvironment and Immune Therapy of Zhejiang Province, Second Affiliated Hospital, Zhejiang University School of Medicine, Hangzhou 310009, Zhejiang, China.; ^3^Department of Surgical Oncology, Taizhou Hospital of Zhejiang Province Affiliated to Wenzhou Medical University, Taizhou 317000, Zhejiang, China.; ^4^Department of Breast Surgery, First Affiliated Hospital of Wenzhou Medical University, Wenzhou 325000, Zhejiang, China.

**Keywords:** Antibody-drug conjugates, breast cancer, treatment resistance, combination strategies

## Abstract

Antibody-drug conjugates (ADCs), inspired by Paul Ehrlich’s “magic bullet” concept to target cancer cells with cytotoxic drugs while sparing healthy cells, represent a transformative approach in breast cancer therapy. From early agents (e.g., gemtuzumab ozogamicin) to second-generation trastuzumab emtansine (T-DM1) and third-generation trastuzumab deruxtecan (T-DXd)/disitamab vedotin (RC48), ADCs have demonstrated significant clinical benefits, including improved progression-free survival (PFS) and overall survival (OS) in breast cancer, with several approved for clinical use. Ongoing preclinical and clinical studies are rigorously exploring ADC combinations with molecular targeted agents, chemotherapy, and immunotherapy. However, *de novo* and acquired resistance remains a critical barrier to maximizing therapeutic efficacy. This review summarizes ADC mechanisms and clinical outcomes in breast cancer, explores resistance mechanisms, and dissects the biological rationale for combination strategies, aiming to identify novel payloads that enhance patient outcomes.

## INTRODUCTION

Antibody-drug conjugates (ADCs) have emerged as a transformative class of therapies in the treatment of breast cancer, achieving enhanced precision and reduced off-target toxicity compared to conventional chemotherapy^[1]^. Notably, their therapeutic potential extends across a range of human epidermal growth factor receptor 2 (HER2) subtypes, encompassing HER2-positive, HER2-low, and even HER2-negative breast cancers. To date, three ADCs have been formally approved for clinical use in breast cancer: trastuzumab emtansine (T-DM1), trastuzumab deruxtecan (T-DXd), and sacituzumab govitecan (SG).

The efficacy of T-DM1 is firmly established through pivotal trials. The EMILIA study demonstrated that T-DM1 significantly outperforms the combination of lapatinib and capecitabine in terms of progression-free survival (PFS) and overall survival (OS)^[2]^. The HER2CLIMB-02 study has shown that the combination of T-DM1 and tucatinib significantly enhances PFS among patients with HER2-positive advanced breast cancer, while OS data remain under ongoing evaluation^[3]^. The KATHERINE study demonstrated that T-DM1, when administered as adjuvant therapy to patients with HER2-positive breast cancer who did not achieve pathological complete response (pCR) following neoadjuvant therapy, significantly enhanced invasive disease-free survival (iDFS) compared to trastuzumab. This improvement translated to a notable reduction in the risk of disease recurrence and death^[4]^. T-DM1 achieved sustained improvements in both OS and iDFS among patients with HER2-positive early breast cancer who had residual invasive disease following neoadjuvant therapy (89.1% *vs.* 84.4%). With a median follow-up of 8.4 years, T-DM1 continued to demonstrate superior iDFS compared to trastuzumab, with a hazard ratio (HR) of 0.55 (95%CI, 0.44 to 0.66). The 7-year iDFS rates were 80.8% for T-DM1 and 67.1% for trastuzumab. Additionally, T-DM1 was associated with a significantly lower risk of death compared to trastuzumab (HR = 0.67; 95%CI: 0.51 to 0.87; *P* = 0.003)^[5]^.

Unlike T-DM1’s antimicrotubule payload, T-DXd features a topoisomerase I inhibitor, which enhances its efficacy. The phase III DESTINY-Breast 03 trial represented a significant milestone: T-DXd became the first ADC to surpass T-DM1 in treating second-line HER2-positive advanced breast cancer. It demonstrated substantial advantages in PFS and OS among patients who had previously received trastuzumab and taxanes^[6]^. A detailed analysis revealed a median overall survival (mOS) of 52.6 months for T-DXd *vs.* 42.7 months for T-DM1 (HR = 0.73; 95%CI: 0.56-0.94), highlighting the significant survival benefit of T-DXd^[7]^. Importantly, the efficacy of T-DXd was consistently demonstrated across various molecular subtypes, including HER2 gene copy number, PI3K mutations, homologous recombination deficiency (HRD), and BRCA1/2 status. In light of the results from the DESTINY-Breast 04 trial, the 2025 CSCO BC Guidelines now recommend T-DXd as the first-line treatment for advanced HR+/HER2-low breast cancer^[8]^.

Beyond T-DM1 and T-DXd, several emerging ADCs are showing promising potential. ARX788 and A166 are currently under active investigation. Meanwhile, SG, which targets trophoblast cell-surface antigen 2 (TROP2), has significantly transformed the treatment landscape for triple-negative breast cancer (TNBC). The TROPiCS-02 study and the Chinese EVER-132-002 study demonstrated that SG achieved a median PFS of 4.3 to 5.5 months^[9]^. The ASCENT and Chinese bridging studies demonstrated that, compared to chemotherapy, treatment with SG for advanced TNBC resulted in a PFS of approximately 5.6 months. This represents a significant improvement in both median PFS and OS for patients^[10]^.

Based on the phase III TB-01 trial, the FDA has approved Dato-Dx for the treatment of breast cancer. In the trial, 732 patients with HR+/HER2-negative advanced breast cancer who had experienced disease progression on endocrine therapy, for whom further endocrine therapy was unsuitable, and who had received 1-2 lines of prior chemotherapy were randomized to receive either Dato-Dx (365 patients) or investigator’s choice of chemotherapy (ICC) (eribulin, vinorelbine, capecitabine, or gemcitabine) (367 patients). Dato-Dx significantly prolonged PFS compared to chemotherapy, with a blinded independent central review (BICR)-assessed median PFS of 6.9 *vs.* 4.9 months (HR = 0.63; 95%CI: 0.52-0.76; *P* < 0.0001), reducing the risk of disease progression or death by 37%. Consistent PFS benefits were observed across subgroups, regardless of prior CDK4/6 inhibitor (CDK4/6i) use, duration of CDK4/6i therapy (≤ 12 or > 12 months), duration of endocrine therapy (≤ 6 or > 6 months), or presence of brain metastases. In secondary endpoints, Dato-Dx also demonstrated notable short-term efficacy and safety^[11]^. A total of 83 Chinese patients (44 in the Dato-Dx group and 39 in the chemotherapy group) participated in the study, with efficacy and safety outcomes aligning with those observed in the global patient population.

These novel ADCs, distinguished by their diverse antigen targets and unique drug-loading mechanisms, are currently being evaluated in extensive clinical trials that include both monotherapy and combination regimens. These investigations are designed to expand therapeutic options for breast cancer patients and drive advancements in treatment paradigms. However, researchers are not content with this progress alone. On one hand, they strive to maximize the antitumor efficacy of ADCs; on the other hand, they seek to address resistance to ADC monotherapy. As a result, combination strategies have emerged as a critical area of ongoing exploration^[12]^.

A paramount challenge in the treatment of breast cancer is drug resistance. This resistance manifests in two primary forms: intrinsic resistance, where tumors are inherently unresponsive to therapy, and acquired resistance, which develops over time under selective pressure from treatment^[13]^. Tumor heterogeneity further complicates this issue: subpopulations of cancer cells with distinct genetic and phenotypic traits can survive initial therapy, driving clonal evolution. This dynamic process allows tumors to adapt, evade therapeutic effects, and ultimately relapse with a more aggressive and treatment-resistant phenotype^[14]^.

Another critical challenge is the incomplete understanding of the mechanisms underlying drug resistance. While progress has been made in identifying genetic markers and molecular pathways associated with resistance, substantial gaps in knowledge remain^[15]^.

In conclusion, cancer drug resistance is a multifaceted problem that requires a comprehensive strategy. This strategy must address three key dimensions: elucidating the biological underpinnings of resistance, accelerating the clinical translation of genomic data, and developing innovative therapeutic approaches to address tumor heterogeneity. Overcoming these challenges is essential for improving treatment outcomes and prolonging OS in cancer patients.

## THE MECHANISM AND DRUG RESISTANCE OF ADCS

### The structure of ADCs and its mechanism

ADCs bind to antigens on the surface of cancer cells and are internalized via receptor-mediated endocytosis. Subsequently, they follow the endosome-lysosome pathway to release their cytotoxic payload, which induces DNA damage or disrupts microtubule structure, ultimately leading to cancer cell death^[16]^.

**Figure 1:**
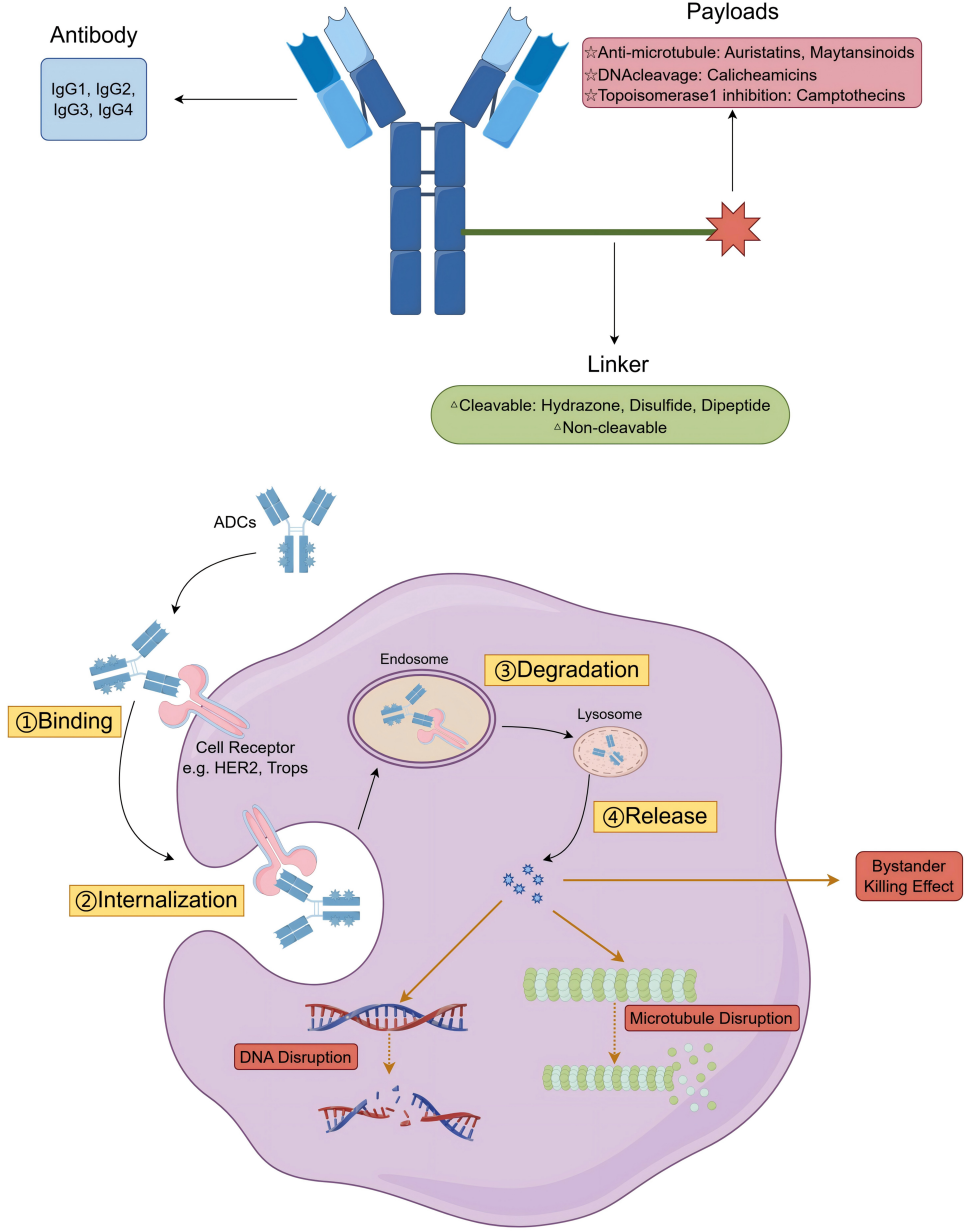
The structure of ADCs and its mechanism. (A) Basic structure of ADCs; (B) Anticancer Mechanism of ADCs. ADCs: Antibody-drug conjugates.

ADCs consist of three essential components: monoclonal antibodies, linkers, and cytotoxic drug payloads [Figure 1A]. Each component plays a critical role in the overall function and pharmacokinetics of ADCs, contributing to their complex behavior *in vivo*^[17]^. The monoclonal antibodies in ADCs must exhibit high affinity and selectivity for their specific targets, recognizing overexpressed antigens (neoantigens) at the tumor site while minimizing off-target delivery of the cytotoxic payload to healthy tissues^[18]^. Humanized monoclonal antibodies are essential for ADCs. These antibodies are classified into two types based on their ability to mediate endocytosis: endocytic antibodies, which facilitate the internalization of the ADC into cancer cells, and non-endocytic antibodies, which do not promote this internalization process. The choice between these types is crucial for optimizing the delivery and efficacy of ADCs^[19]^.

The selection of linkers in ADCs should be guided by the mechanism of action of the antibody, while minimizing potential chemical modifications to the drug payload. Linkers must also exhibit sufficient stability in a biological environment to ensure reliable drug delivery. Linkers can be broadly classified into two categories: cleavable and non-cleavable. Cleavable linkers are designed to release the cytotoxic payload in response to specific conditions within the tumor microenvironment, such as changes in pH or the presence of specific enzymes. This targeted release mechanism aims to enhance the therapeutic effect while minimizing systemic exposure to the drug. In contrast, non-cleavable linkers offer superior plasma stability compared to cleavable linkers, ensuring that the drug payload remains attached to the antibody until it reaches the target cancer cells. This enhanced stability reduces the risk of off-target toxicity and improves the overall tolerability and efficacy of the ADC. The choice between cleavable and non-cleavable linkers is critical, as it directly impacts the pharmacokinetics, safety, and therapeutic efficacy of the ADC. Non-cleavable linkers are often preferred for their ability to provide greater stability and tolerability, thereby reducing off-target toxicity and enhancing the overall therapeutic profile of the ADC^[20]^.

The drug payload in ADCs must meet several critical criteria: it should have good solubility in aqueous solutions, exhibit significantly higher cytotoxic activity compared to standard chemotherapy drugs, and induce cancer cell death through apoptotic mechanisms. Additionally, it must possess appropriate functional groups to facilitate binding with the antibody. Microtubule-targeting drugs are among the most widely used drug payloads in ADCs due to their well-established mechanisms of action and efficacy. Another important category of drug payloads includes DNA-damaging agents, which are often more potent than microtubule inhibitors. This higher potency makes them particularly suitable for targeting antigens that are expressed at low levels on tumor cells. Importantly, these DNA-damaging agents can induce apoptosis in both dividing and non-dividing cancer cells, thereby expanding the therapeutic window and enhancing the overall efficacy of ADCs^[21]^. Other mechanisms underlying ADC drug payloads include direct induction of apoptosis, spliceosome inhibition, and RNA polymerase inhibition, among others. Current ADC research faces several key challenges, including the selection of appropriate target cells, the nature and expression levels of tumor antigens, the structure and stability of the antibodies, linker chemistry, and the choice of cytotoxic payload^[22]^ [Figure 1B].

### Molecular mechanisms of resistance to ADCs

**Figure 2:**
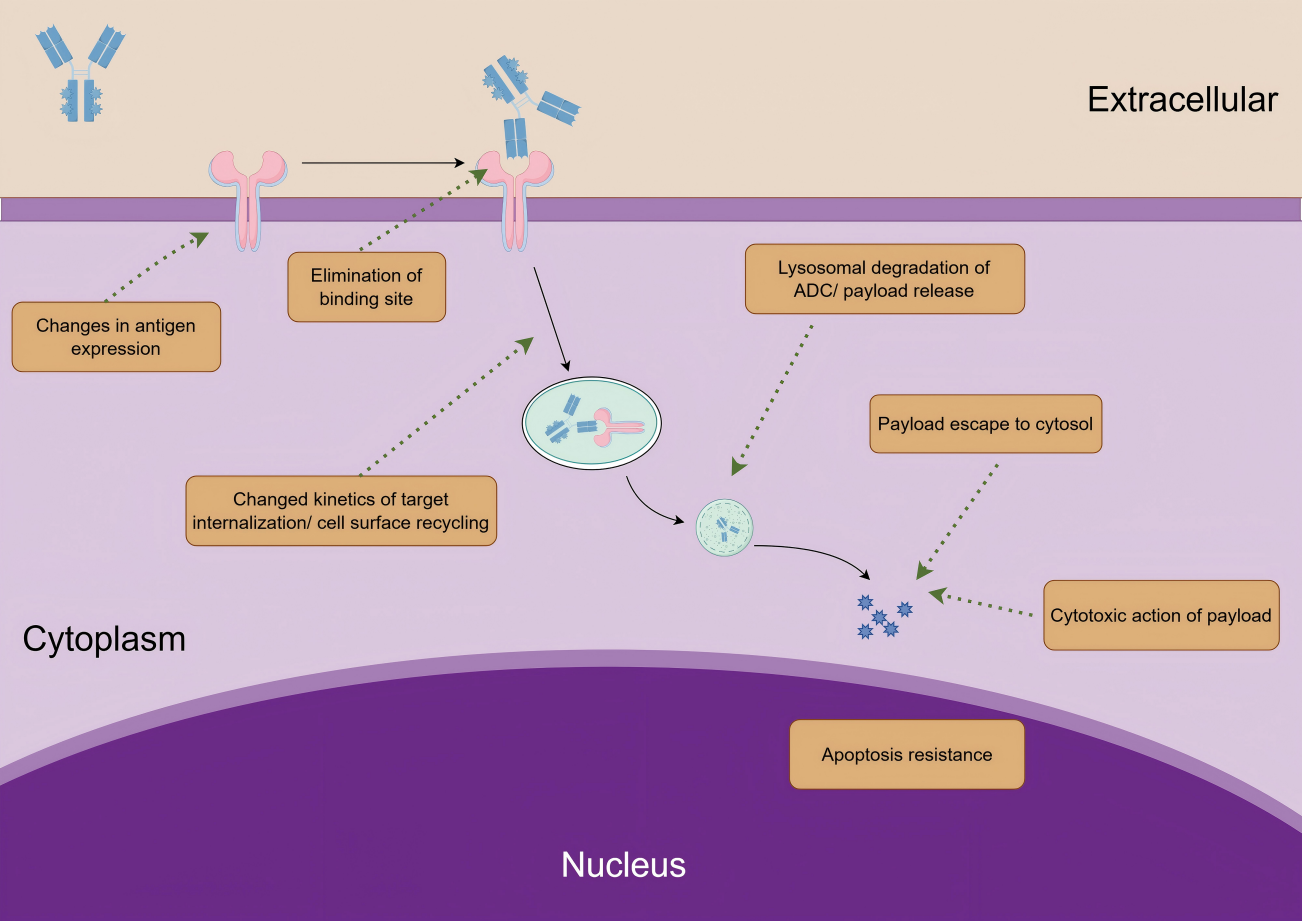
Overview of mechanisms of ADC resistance. ADC: Antibody-drug conjugate.

Given the complexity of ADCs, which consist of multiple components, resistance to these therapies is highly complex and may involve multiple resistance mechanisms for the same ADC^[23]^. Any abnormality at any stage of the ADC process can lead to drug resistance. These abnormalities may include changes in antigen expression, development of tolerance to the cytotoxic payload, obstruction of drug internalization and transport, lysosomal dysfunction, overexpression of drug efflux pump proteins, and activation of bypass signaling pathways [Figure 2].

#### Changes in antigen expression

The internalization of ADC-antigen complexes is essential for the effective delivery of cytotoxic payloads in many ADC drugs, a process that relies heavily on the overexpression of internalizing antigens on the surface of tumor cells. A reduction in the expression of these tumor cell surface antigens that bind to ADCs can result in a scarcity of recognizable targets, thereby impeding the drugs from exerting their intended cytotoxic effects. For HER2-targeting ADCs, the most significant mechanisms of resistance are often associated with alterations in the expression or presentation of the HER2 antigen itself^[24]^. Loganzo *et al.* discovered that exposing breast cancer cells to trastuzumab-maytansinoid ADC (TM-ADC) can result in reduced HER2 protein levels. This reduction may contribute to drug resistance and ultimately lead to treatment refractoriness^[25]^. Furthermore, research has demonstrated that the heterogeneity of tumor antigen expression can significantly impact the efficacy of ADCs. In the KRISTINE trial, 80% of patients treated with T-DM1 plus pertuzumab exhibited HER2 intratumoral heterogeneity (ITH), which was associated with local disease progression prior to surgery. In contrast, only 15% of patients without local disease progression showed this phenomenon. Similarly, in the Phase 2 DAISY study, T-DXd was evaluated in patients with advanced breast cancer characterized by varying levels of HER2 expression. The study found that higher HER2 expression levels were associated with higher ORR and longer PFS^[26]^.

#### Elimination of binding site by alternative splicing and mutation or masking of binding site

The truncation of the antigen extracellular domain or the masking of extracellular matrix components may contribute to the resistance mechanism of HER2 to trastuzumab. In a subgroup of tumors that overexpress HER2, a truncated receptor variant, p95HER2, which retains kinase activity, is expressed. This variant has been shown to be directly associated with trastuzumab resistance^[27]^. The masking of antigen binding sites can result in insensitivity to antibodies. For instance, the presence of HER3 ligands can interfere with the presentation of HER2 antigens, thereby diminishing the therapeutic efficacy of T-DM1^[28]^. Furthermore, the Trop-2 TACSTD2 T256R gene mutation has been shown to directly confer resistance to SG^[29]^. In TNBC, concurrent genomic alterations in antigens and cytotoxic payload targets can mediate the development of acquired resistance to SG^[30]^.

#### Changed kinetics of target internalization/cell surface recycling

Receptors facilitate the entry of ADCs into cells through endocytosis, a process that can occur via multiple internalization pathways. In solid tumors with a dense extracellular matrix, targeting internalizing antigens on the surface of cancer cells presents significant challenges. Factors such as changes in the kinetics of target internalization, alterations in surface cycling, or disruptions in intracellular transport can all interfere with this process. For example, clathrin-mediated endocytosis (CME), caveolin-mediated endocytosis, and clathrin-caveolin-independent endocytosis are all potential pathways for ADC internalization. Sung *et al.* reported that N87-TM cells (a variant of N87 cells resistant to T-DM1) exhibited increased intracellular caveolin levels and utilized caveolin-mediated endocytosis to internalize trastuzumab ADC. Since T-DM1 is metabolized within lysosomes, the reduced lysosomal co-localization of trastuzumab in N87-TM cells results in inefficient delivery of T-DM1 to lysosomes, thereby diminishing its therapeutic efficacy^[31]^. Endophilin A2, encoded by the *SH3GL1* gene, has been demonstrated to promote HER2 internalization and enhance the sensitivity of breast cancer cells to trastuzumab-based therapies. Conversely, knockdown of SH3GL1 in tumor cells results in decreased HER2 internalization and significantly diminishes the cytotoxic effects mediated by T-DM1^[32]^.

#### Lysosomal degradation of ADC/payload release

Lysosomal degradation is a critical process for effectively breaking down and releasing the cytotoxic payload from ADCs. However, this process can be disrupted by factors such as reduced lysosomal acidification or decreased proteolytic activity. For ADCs to exert their therapeutic effects, they must reach lysosomes, where the cytotoxic agents are released through chemical or enzymatic cleavage.

However, an increase in lysosomal pH can significantly inhibit the activity of lysosomal proteolytic enzymes. Research has demonstrated that lysosomes in a higher pH environment exhibit impaired proteolytic activity, which can prevent the adequate cleavage of T-DM1. This disruption in the degradation process ultimately leads to resistance to ADC therapy^[33]^.

#### Payload escape to the cytosol

This resistance mechanism is associated with the transport of the cytotoxic payload from the lysosomal lumen to the cytoplasm, a process that is particularly critical for non-cleavable linkers. These linkers often require a specific transporter to deliver the payload to the cytoplasm, where it can exert its cytotoxic effects. It has been discovered that SLC46A3 functions as the direct transporter of DM1 metabolites from the lysosome to the cytoplasm. Consequently, the loss of SLC46A3 expression represents one of the key mechanisms underlying resistance to T-DM1^[34]^. Abnormal expression of drug efflux transporters, particularly ATP-binding cassette (ABC) transporters, can lead to the transport of the cytotoxic payload outside the cells, thereby preventing it from exerting its full cytotoxic effect. In cells with higher expression of ABC transporters, the half-maximal inhibitory concentration (IC50) ratio of DXd to exatecan is elevated, indicating a greater difference in activity between the two compounds. This phenomenon underscores the significant role of ABC transporters in mediating drug resistance. The addition of ABC transporter inhibitors has been shown to significantly enhance the activity of DXd and SN-38, thereby overcoming this resistance mechanism^[35]^.

#### Cytotoxic action of payload

Mutations in payload targets are a significant contributor to ADC resistance mechanisms. SG is composed of the anti-TROP2 antibody hRS7 conjugated to a topoisomerase 1 (TOP1) inhibitor payload. Through RNA and whole-exome sequencing, Coates *et al.* discovered that complete loss of TROP2 expression may serve as an important predictor of primary resistance to SG. Furthermore, within the same patient, different metastatic lesions may develop acquired resistance to SG. This resistance is characterized by mutually exclusive somatic mutations in genes encoding TROP2 (the antibody target) and TOP1 (the payload target)^[30]^.

Studies have found that T-DM1 can bind to HER2 and be internalized in both T-DM1-resistant and -sensitive cells. However, T-DM1 induces Cyclin B1 aggregation in sensitive cells but fails to do so in resistant cells. Elevating Cyclin B1 levels can partially restore sensitivity in resistant cells. The inability of T-DM1 to induce Cyclin B1 aggregation contributes to acquired resistance in HER2-positive breast cancer^[36]^.

Another study indicated that the expression of the mitotic kinase polo-like kinase 1 (PLK1) is elevated in T-DM1-resistant models compared to parental cell lines. In preclinical models, inhibition of PLK1 using volasertib has been shown to reverse T-DM1 resistance at both the genomic and pharmacological levels^[37]^. In the DAISY study, among patients treated with T-DXd, 20% of patients carried mutations in the *SLX4* gene (SLX4 Structure-Specific Endonuclease Subunit), which encodes a DNA repair protein. These mutations were associated with potential molecular abnormalities linked to resistance. Researchers conducted *in vitro* cell experiments and proposed that SLX4 mutations may induce secondary resistance to DXd^[26]^.

#### Apoptosis resistance

Dysregulation of the apoptosis pathway, characterized by the absence of pro-apoptotic proteins Bak and Bax or the overexpression of anti-apoptotic proteins B-cell lymphoma-2 (Bcl-2) and Bcl-x, can also contribute to resistance to ADCs^[38]^. Activation of the PI3K/AKT/mTOR pathway may lead to decreased sensitivity to ADCs, reduced effectiveness of cytotoxic payloads, and enhanced cell survival. Additionally, loss of phosphatase and tensin homolog deleted on chromosome ten (PTEN) or excessive activation of phosphatidylinositol-4,5-bisphosphate 3-kinase catalytic subunit alpha (PIK3CA) can result in decreased sensitivity to trastuzumab through the activation of the phosphoinositide 3-kinase-Akt (PI3K/AKT) signaling pathway^[39]^. However, exploratory biomarker analysis from the EMILIA trial revealed that, compared to lapatinib and capecitabine, T-DM1 not only extended OS and PFS in metastatic breast cancer (mBC) patients who had previously received trastuzumab and taxanes, but also demonstrated comparable efficacy in tumors with PIK3CA mutations and those with wild-type PIK3CA^[40]^. Multiple mechanisms can influence the cytotoxic action of the payload. Additionally, some mechanisms can interfere with apoptosis-induced tumor cell death. Other mechanisms mediating T-DXd resistance in breast cancer include the suppression of crVDAC3, which leads to a dramatic increase in excessive ROS (reactive oxygen species) levels and labile iron pool accumulation. The inhibition of crVDAC3 induces ferroptosis in breast cancer cells by reducing HSPB1 expression^[41]^.

## THE THEORETICAL BASIS OF ADC-BASED COMBINATION THERAPY

ADCs hold great promise for the treatment of breast cancer. However, the duration of objective response or clinical benefit achieved with ADC monotherapy is often limited due to the emergence of resistance mechanisms, a challenge commonly encountered with most cytotoxic agents. Maximizing the antitumor efficacy of ADCs while addressing resistance issues associated with monotherapy has thus emerged as a critical research direction^[42]^. Consequently, the combination of ADCs with other anticancer therapies - such as chemotherapy, molecularly targeted agents, and immunotherapy - is being actively explored in both preclinical models and clinical trials^[43]^. Meta-analyses have demonstrated that combination therapies involving ADCs generally exhibit superior efficacy compared to ADC monotherapy^[44]^. Research has highlighted that the optimal combination strategy for ADCs should produce additive or synergistic effects on tumor cells or within the tumor microenvironment, while avoiding overlapping toxicity profiles^[45]^. These benefits can be attributed to several specific mechanisms, which are outlined below.

### Enhancing ADC delivery to tumor tissue

For instance, anti-angiogenic agents can enhance the delivery of ADCs to tumor tissue by promoting the normalization of tumor vasculature, which in turn improves the cytotoxic efficacy of ADCs.

### Regulating antibody target protein expression

Drugs can work synergistically to affect the cell cycle and modulate the expression of surface antigens. Increasing the expression of tumor cell surface antigens can enhance the binding affinity of antibodies to their targets. Additionally, drugs that promote antigen conversion or degradation may facilitate the uptake of ADCs, leading to more efficient cleavage and release of the payload, thereby enhancing cytotoxicity.

### Enhancing payload activity and synthetic lethality

Molecularly targeted drugs can be combined with ADCs to simultaneously block multiple oncogenic pathways or to doubly inhibit a specific pathway, thereby more effectively suppressing downstream signaling. Other drugs that exert synergistic effects through complementary mechanisms or synthetic lethality can enhance the activity of the payload, leading to more potent antitumor effects.

### Promoting antitumor immunity

Immunotherapy has the potential to augment the antitumor immunity induced by ADCs. This can be achieved by enhancing antibody-dependent cellular cytotoxicity (ADCC) or by improving cell-mediated tumor recognition and immune effector functions, thereby amplifying the overall antitumor response.

## ADCS COMBINED WITH CHEMOTHERAPY

Chemotherapy and ADCs can exert synergistic effects through multiple mechanisms, such as targeting different stages of the cell cycle or modulating the expression of tumor cell surface antigens^[44]^.

### Combined with taxanes

A study evaluating T-DM1 in combination with docetaxel (with or without pertuzumab) for HER2-positive breast cancer demonstrated that the objective response rate (ORR) of combination therapy was 47.8%, with a median PFS of 7.4 months. However, grade 3 or higher adverse events and dose-limiting toxicities (DLTs) were observed in approximately 80% of patients with mBC^[46]^. Another study evaluated the maximum tolerated dose (MTD) of X in combination with T-DM1 for patients with HER2-positive mBC and demonstrated that the combination of T-DM1 and X at 750 mg/m^2^ showed encouraging activity. However, due to DLTs, a dose de-escalation cohort was deemed necessary^[47]^. A study evaluating the combination of T-DM1, lapatinib, and nab-paclitaxel in patients with metastatic HER2-overexpressing breast cancer reported that 12 patients (85.7%) achieved an objective response, including 6 complete responses (CR) and 2 partial responses (PR). The combination therapy of T-DM1, lapatinib, and nab-paclitaxel was relatively well-tolerated and demonstrated significant antitumor activity^[48]^. Neoadjuvant treatment with T-DM1, lapatinib, and nab-paclitaxel demonstrated superior efficacy compared to standard treatment, particularly in the ER-positive cohort of early-stage HER2-positive breast cancer^[49]^.

### Combined with anthracyclines

The Phase 1b portion of the Thelma study evaluated the efficacy of combining T-DM1 with anthracycline drugs for treating HER2-positive advanced breast cancer. The ORR for various dose combinations was approximately 40%. While the combination of T-DM1 and non-pegylated liposomal doxorubicin (NPLD) was found to be safe, it did not appear to enhance the antitumor activity of T-DM1^[50]^.

While evidence suggests that combining ADCs with chemotherapy may enhance antitumor activity, the additive toxicity remains a significant challenge, with over half of patients requiring dose reductions or discontinuation of treatment. Future combination therapies will need to ensure adequate tolerability, optimize ADC selection, and carefully choose appropriate tumor types and combination partners. For high-risk patients, such as those who do not respond well to standard neoadjuvant therapy or those who fail to achieve a pathological complete response (non-pCR), the potential benefits of combination therapy are particularly promising.

### ADCs combined with targeted therapy

ADCs offer superior therapeutic effects compared to traditional chemotherapy drugs and can be effectively integrated into various combination strategies. This approach helps to overcome drug resistance and clonal heterogeneity, resulting in more robust inhibition of cancer gene-dependent signaling pathways. Moreover, ADCs enhance the expression of surface antigens, thereby increasing the sensitivity of low-antigen-expressing tumors, and they also modulate the tumor microenvironment.

Clinical trials such as KAITLIN, KRISTINE, and MARIANNE were designed based on the preclinical synergistic antitumor activity observed with the combination of T-DM1 and pertuzumab. However, in both neoadjuvant and metastatic settings, this combination did not demonstrate superior efficacy compared to the regimen of paclitaxel, trastuzumab, and pertuzumab. This was particularly evident in patients with low HER2 expression or high HER2 heterogeneity. Additionally, the safety profile of the T-DM1 and pertuzumab combination was not significantly different from that of traditional therapies^[51-53]^. The DESTINY-Breast 07 study, designed to compare the efficacy of T-DXd monotherapy with the combination of T-DXd and pertuzumab, demonstrated similar ORR between the two groups (84% *vs.* 76%). The 12-month PFS rates were 80.8% for T-DXd monotherapy and 89.4% for the combination therapy, with no new safety signals identified. The use of pertuzumab in combination with T-DXd can enhance the internalization of T-DXd, potentially leading to more effective antitumor activity^[54]^. Currently, the DB09 trial is exploring the efficacy of this combination strategy in first-line HER2-positive patients.

The TEAL study demonstrated that, compared to the standard THP combination regimen, the combination of T-DM1, lapatinib, and nab-paclitaxel significantly increased the pCR to 100%, compared with 62.5% in the standard regimen, particularly in the hormone receptor-positive subgroup (pCR 100% *vs.* 25%). In patients with advanced disease who had progressed after prior treatment with trastuzumab and taxanes, the ORR was 47%, including a response rate of 36% among those with brain metastases^[49]^. Newer ADCs and tyrosine kinase inhibitors (TKIs) may offer improved outcomes. Consequently, additional Phase III clinical trials have been initiated to explore the combination of tucatinib with T-DM1 (HER2CLIMB-02) and T-DXd (HER2CLIMB-04)^[55]^. The addition of tucatinib to T-DM1 significantly improved PFS in patients with previously treated HER2-positive locally advanced or metastatic breast cancer (LA/mBC). The median PFS was 9.5 months with the combination therapy, compared to 7.4 months with T-DM1 alone (HR = 0.76; *P* = 0.0163). In patients with brain metastases, the median PFS was 7.8 months with the combination therapy, compared to 5.7 months with T-DM1 alone (HR = 0.64)^[55]^. The adverse events observed were consistent with those previously reported for tucatinib and T-DM1. The HER2CLIMB-04 Phase II clinical trial is currently evaluating the clinical efficacy of tucatinib in combination with T-DXd in patients with HER2-positive, unresectable, LA, or mBC. The results of this trial are highly anticipated.

The TBCRC 022 study represents the first assessment of the efficacy and safety of combining neratinib with T-DM1 in patients with HER2-positive breast cancer and brain metastases. This study is also the first to evaluate a T-DM1-containing regimen following progression on T-DM1 alone, suggesting potential synergistic effects with neratinib. The safety and tolerability profiles observed thus far are deemed acceptable^[56]^.

A Phase Ib study indicated that the combination of T-DXd and nivolumab exhibited antitumor activity in patients with HER2-positive mBC or metastatic urothelial cancer (mUC). The confirmed objective response rate (cORR) was 65.6% in patients with HER2-positive mBC and 50.0% in those with HER2-low mBC. The combination therapy demonstrated a manageable safety profile, consistent with the known safety data for T-DXd^[57]^. The DESTINY-Breast07 study, which explored the safety and antitumor activity of T-DXd alone or in combination with pertuzumab as first-line (1L) treatment in patients with HER2-positive mBC, reported a confirmed ORR of 77.3% with T-DXd monotherapy and 82.0% with the combination of T-DXd and pertuzumab. The PFSmrate at 12 months was 77.3% with T-DXd alone and 89.4% with the combination therapy. Safety profiles were consistent with the known profiles for T-DXd and pertuzumab^[54]^. The TROPHY trial, which is currently in progress, is exploring the use of T-DXd in combination with pyrotinib as a first-line treatment for patients with HER2-positive unresectable or mBC^[58]^.

Preclinical data indicate that SG synergizes with poly (ADP-ribose) polymerase inhibitors (PARPi) to induce DNA damage and cell death. Combining SG-mediated TOP inhibition with the synthetic lethality of PARPi offers additional benefits in TNBC, regardless of BRCA1/2 status. SG combined with three different PARPi variants resulted in synergistic growth inhibition, increased double-strand (ds) DNA breaks, and S-phase cell cycle accumulation. In mice bearing BRCA1/2-mutated TNBC tumors, the combination of SG with olaparib or talazoparib enhanced antitumor effects and delayed tumor progression. In mice with BRCA1/2 wild-type tumors, SG plus olaparib demonstrated significant antitumor and survival benefits, with well-tolerated combination therapies and no major hematologic changes^[59]^. In a Phase 2 study, the combination of SG with PARPi demonstrated preliminary evidence of efficacy, with a cORR of 30.1% in patients with metastatic triple-negative breast cancer (mTNBC)^[60]^. Clinical studies exploring the combination of other PI3Kα inhibitors with ADCs are currently in progress.

Given that the malignant transformation of breast epithelial cells driven by HER2 relies on cyclin D1, the combination of CDK4/6 inhibitors with T-DM1 has been explored in patients with HER2 resistance. A Phase 1 study evaluating the combination of T-DM1 and palbociclib demonstrated an ORR of only 33%, with a median PFS of 6 months. Additionally, the incidence of Grade 3 hematologic toxicity exceeded 10%^[61]^. Similarly, the PFS of T-DM1 in combination with ribociclib was 10.4 months^[62]^. This phenomenon may be related to CDK4/6 inhibitors preventing tumor cells from entering the S/M phase, thereby reducing the efficacy of T-DM1.

## ADCS COMBINED WITH IMMUNOTHERAPY

Emerging evidence indicates that ADCs may potentiate the efficacy of immunotherapy agents. The underlying mechanisms include inducing immunogenic cell death, promoting dendritic cell maturation, enhancing T lymphocyte infiltration, and augmenting immune memory. Additionally, ADCs can upregulate the expression of immune regulatory proteins, such as programmed death ligand 1 (PD-L1) and major histocompatibility complex (MHC) molecules^[63]^. However, current combinations of ADCs with immunotherapy primarily focus on PD-1/PD-L1 and cytotoxic T-lymphocyte-associated protein 4 (CTLA-4). In breast cancer, the exploration of these combinations is particularly limited to PD-1/PD-L1.

The KATE2 study, which was the first to explore the combination of ADCs and immunotherapy, demonstrated no significant difference in median PFS between the T-DM1 plus atezolizumab group and the control group (8.2 *vs.* 6.8 months, *P* = 0.33). However, the combination reduced the risk of progression by 60% in the PD-L1-positive subgroup. Further studies are warranted to fully elucidate the potential benefits of this combination^[64]^. Studies have suggested that combinations of atezolizumab with ADCC agents or ADCs are generally well-tolerated. Further biomarker analyses have demonstrated that these combinations can promote the activation of the adaptive immune system within the tumor microenvironment^[65]^. The DS8201-A-U105 trial indicated that the combination of T-DXd and nivolumab did not enhance efficacy in patients with HER2-positive mBC. The safety profile of the combination therapy was comparable to that of monotherapy^[66]^. The BEGONIA study indicated that the combination of Dato-DXd and durvalumab achieved favorable response rates and demonstrated a manageable safety profile as a first-line treatment for advanced or metastatic triple-negative breast cancer (a/mTNBC). The adverse events observed were manageable, supporting the need for further investigation into this therapeutic approach^[67]^. Additionally, research into the combination of SG and pembrolizumab for the treatment of both early and advanced breast cancer is ongoing.

Current clinical data suggest that combining ADCs with immunotherapy can yield favorable response rates. This combination therapy appears to be particularly feasible for elderly and frail patients who are at higher risk of experiencing chemotherapy-related toxicities. Moreover, there is a notable scarcity of publicly available clinical data regarding the combination of ADCs with immunotherapies beyond anti-PD-1/PD-L1/CTLA-4 antibodies.

## ADCS COMBINED WITH ENDOCRINE THERAPY

The WSG ADAPT study indicates that the pCR rate is significantly higher with T-DM1 monotherapy or when T-DM1 is combined with endocrine neoadjuvant therapy, compared to trastuzumab plus endocrine therapy. However, no significant difference in the pCR rate was observed between the T-DM1 monotherapy group and the group receiving T-DM1 in combination with endocrine therapy^[68]^. In the KRISTINE trial, patients with tumors that were estrogen receptor-positive, progesterone receptor-positive, or both received adjuvant endocrine therapy. No significant differences between the two groups were observed in terms of grade ≥ 3 adverse events (26.0% *vs.* 24.9%)^[4]^. In the DB-08 study, the safety profile of T-DXd in combination with endocrine therapy is comparable to that of T-DXd monotherapy. The combination of T-DXd with anastrozole or fulvestrant demonstrates positive antitumor effects in first- or second-line treatment for HR-positive mBC with low HER2 expression^[69]^.

The side effects associated with endocrine therapy are minimal. This minimal side effect profile gives clinicians greater confidence in incorporating ADC drugs into endocrine therapy regimens. Currently published clinical studies rarely feature cases of T-DM1 combined with endocrine therapy. However, research on the combination of other ADC drugs with endocrine therapy is still ongoing.

## ADCS COMBINED WITH RADIOTHERAPY

Radiotherapy is another crucial treatment modality for tumors, particularly for patients undergoing breast-conserving surgery for breast cancer, those with axillary lymph node metastasis, and those with brain metastases. Although numerous clinical studies are currently underway, their results have not yet been published. An international multicenter retrospective study (the TENDANCE study) was designed to evaluate the safety and efficacy of combining T-DXd with radiotherapy. The study demonstrated that 22.2% of patients achieved a CR and 77.8% had either a PR or stable disease, with a median follow-up of 9 months. These findings suggest that the combination of T-DXd and radiotherapy is feasible and associated with a manageable toxicity profile^[70]^. In the KATHERINE trial involving T-DM1, patients who underwent postoperative breast radiotherapy were included. However, no specific radiotherapy-related toxicities were reported, nor was there an observed increase in pulmonary toxicity. These findings suggest that the use of T-DM1 during adjuvant breast radiotherapy is relatively safe. Local radiotherapy for breast cancer can be concurrently administered with T-DM1-based targeted therapy. However, compared with whole brain radiotherapy, the combination of T-DM1 with stereotactic radiosurgery for brain metastases significantly increases the risk of symptomatic radiation necrosis in the later stages^[71]^. Overall, there is currently insufficient evidence to assess the safety of combining whole brain radiotherapy, palliative extracranial radiotherapy, or stereotactic radiotherapy with T-DM1. Therefore, concurrent use of T-DM1 targeted therapy with whole brain radiotherapy or stereotactic radiosurgery for brain metastases is not currently recommended. Clinical studies of T-DXd have not reported adverse events related to concurrent radiotherapy, including an increased risk of interstitial lung disease. Thus, there is currently a lack of safety data regarding the use of T-DXd in combination with radiotherapy. More data are needed in this area.

## CONCLUSION

ADCs have demonstrated their potent antitumor effects in the treatment of breast cancer in recent years, while continuously expanding their indications.

### Clinical translation challenges

The limitations and challenges of ADC drugs include their complex pharmacokinetic profiles and unavoidable adverse effects. Other challenges involve optimizing tumor targeting, linker stability, and payload release, as well as addressing the issue of drug resistance. Additionally, novel ADCs are constantly being developed [Table 1]. Therefore, the prognosis of breast cancer is expected to be further improved with the aid of ADCs. Maximizing the efficacy of ADCs is currently a hotspot in this field, with research directions focusing on expanding the population that can benefit, addressing resistance, and identifying the best partners to establish rational combination strategies. The latter is particularly eye-catching. Our review systematically summarizes the current resistance mechanisms of ADCs and the prospects for combination therapies, with some studies already showing promising trends of synergistic effects, reduced resistance, and controllable safety [Table 2]. Moreover, the multifunctionality of antibodies, exploration of new antigens, screening of novel cytotoxic drugs, and increasingly complex integration methods are important directions for the future development of ADCs.

**Table 1:** Primary specifics of ADCs currently approved worldwide in treating breast cancer

**Drug name**	**Target**	**Linker**	**Payload**	**Indications**	**Year of approval**	**Clinical trial**	**Efficacy**	**Regimen**
Kadcyla (ado-T-DM1)	HER2 IgG1	SMCC	DM1	HER2+metastatic BC previously treated with trastuzumab & a taxane/HER2+early BC after neoadjuvant taxane & trastuzumab-based treatment	2013/2019	EMILIA trial	T-DM1 *vs.* capecitabine- lapatinib; mOS 30.9 *vs.* 25.1 months (HR = 0.65; 95%CI: 0.64-0.88); T-DM1 *vs.* trastuzumab; 3 years iDFS 88.3% *vs.* 77% (HR = 0.5; 95%CI: 0.39-0.64)	3.6 mg/kg, Q3W
Enhertu (fam-T-DXd-nxki)	HER2 IgG1	GGFG	DXd	Unresectable or metastatic HER2+BC after 2 or more anti-HER2 regimens/LA or metastatic HER2+gastric or gastroesophageal junction adenocarcinoma after a trastuzumab-based regimen	2019/2021	DESTINY-BREAST03 trial	Single-arm ORR = 61.4%/T-DXd *vs.* CT, ORR: 51% *vs.* 14%	6.4 mg/kg, Q3W
Trodelvy (SG-hziy)	TROP2 IgG1	CL2A	SN38	LA or metastatic TNBC after at least two prior therapies	2020	ASCENT trial	SG *vs.* CT; mPFS 5.6 *vs.* 1.7 months (HR = 0.41; 95%CI: 0.32-0.52)	10 mg/kg, days 1 and 8, Q3W
Datopotamab deruxtecan-dlnk (Dato-Dxd, Datroway)	Trop-2 IgG1	GGFG	DXd	Unresectable or metastatic hormone receptor-positive, HER2-negative (lHC 0, lHC1+ or lHC2+/lSH-) breast cancer who have received prior endocrine-based therapy and chemotherapy for unresectable or metastatic disease	2025	TROPION-Breast01 trial	Dato-DXd *vs.* CT; mPFS 6.9 *vs.* 4.9 months (HR = 0.63; *P* < 0.0001); mOS 18.6 *vs.* 18.3 months	6 mg/kg, Q3W

ADCs: Antibody-drug conjugates; HER2: human epidermal growth factor receptor 2; BC: breast cancer; T-DM1: trastuzumab emtansine; mOS: median overall survival; HR: hazard ratio; iDFS: invasive disease-free survival; T-DXd: trastuzumab deruxtecan; LA: locally advanced; ORR: objective response rate; CT: chemotherapy; SG: sacituzumab govitecan; TROP2: trophoblast cell-surface antigen 2; TNBC: triple-negative breast cancer; mPFS: median progression-free survival.

**Table 2:** Summary of clinical trials investigating the combination of ADCs with other drugs

**Drug**	**NCT number**	**Other name**	**Target**	**Partner drugs**	**Phase**	**Start year**	**Treatment setting**	**Efficacy**	**Ref.**
**Combination of ADCs with chemotherapy**									
T-DM1	NCT00934856		HER2	Docetaxel	Ib/IIa	2015	LABC	Positive	[46]
	NCT01702558	TRAXHER2	HER2	Capecitabine	I	2012	mBC	Negative	[47]
	NCT02073916	Stela	HER2	Lapatinib and nab-paclitaxel	I	2013	mBC	Positive	[48]
	NCT02073487	TEAL	HER2	Nab-paclitaxel and lapatinib	II	2014	Neoadjuvant, BC	Positive	[49]
	NCT02562378	Thelm	HER2	NPLD	I	2015	mBC	Negative	[50]
**Combination of ADCs with targeted therapy**									
T-DM1	NCT01966471	KAITLIN	HER2	Pertuzumab	III	2017	HER2-positive EBC	Negative	[52]
	NCT02131064	KRISTINE	HER2	Pertuzumab	III	2014	stage II to III breast cancer (HER2+)	Negative	[53]
	NCT01120184	MARIANNE	HER2	Pertuzumab	III	2010	mBC	Negative	[68]
	NCT02073487	TEAL	HER2	Lapatinib and nab-paclitaxel	II	2014	Neoadjuvant, BC	Positive	[49]
	NCT03975647	HER2CLIMB-02	HER2	Tucatinib	III	2019	mBC	Positive	[72]
	NCT06083662		HER2	Neratinib	II	2018	BC brain metastases	Positive	[56]
	NCT01976169		HER2	Palbociclib	I/Ib	2014	HER2-positive advanced breast cancer	Negative	[61]
	(NCT02657343		HER2	Ribociclib	1b	2016	HER2-positive mBC	Negative	[62]
T-DXd	NCT04538742	DESTINY-Breast07		Pertuzumab	1b/2	2023	Untreated HER2+ mBC	Positive	[54]
	NCT06245824	TROPHY	HER2	Pyrotinib	Ib/II	2024	Unresectable or mBC	Positive	[58]
SG	NCT04039230		TROP2	Talazoparib	II	2021	mTNBC	Positive	[60]
**Combination of ADCs with immunotherapy**									
T-DM1	NCT02924883	KATE2	HER2	Atezolizumab	II	2016	mBC	Negative	[64]
	NCT02605915		HER2	Atezolizumab	Ib	2015	mBC	Positive	[64]
T-DXd	NCT03523572	DS8201-A-U105	HER2	Nivolumab	Ib	2018	mBC	Positive	[66]
	NCT03742102	BEGONIA	TROP2	Durvalumab	IB/II	2018	mBC	Positive	[67]
**Combination of ADCs with endocrine therapy**									
T-DM1	NCT01779206	WSG-ADAPT-TP	HER2	Tamoxifen/aromatase inhibitor	II	2012	HR+/HER2+ early breast cancer	Positive	[68]
	NCT01772472	KATHERINE	HER2	UnspecifedET	III	2013	Adjuvant BC	Positive	[4]
T-DXd	NCT04556773	DB-08	HER2	Anastrozole or fulvestrant	Ib	2020	mBC	Positive	[69]
**Combination of ADCs with radiotherapy**									
T-DXd	/	TENDANCE	HER2	SRT/3D CRT/IMRT/VMAT	/	2021	mBC	Positive	[70]
T-DM1	Retrospective study	/	HER2	Stereotactic radiosurgery	/	2004-2017	BC	Negative	[71]

ADCs: Antibody-drug conjugates; HER2: human epidermal growth factor receptor 2; LABC: locally advanced breast cancer; mBC: metastatic breast cancer; NPLD: non-pegylated liposomal doxorubicin; T-DM1: trastuzumab emtansine; EBC: early breast cancer; TROP2: trophoblast cell-surface antigen 2; mTNBC: metastatic triple-negative breast cancer; T-DXd: trastuzumab deruxtecan.

New ADCs based on existing targets include SYD985, ARX788, and RC48 targeting HER2, and Dato-DXd targeting TROP2. ADCs based on emerging targets such as human epidermal growth factor receptor 3 (HER3), Nectin-4, Human B7 homolog 3 (B7-H3), and LIV1 are also under development, which are highly anticipated for the future.

### Future research directions

As the number of approved ADCs continues to rise, so does the number entering clinical trials, with new patterns constantly emerging. The future development directions lie in the following aspects:

ADCs are revolutionizing oncology and targeted therapy. In 2024, double-payload ADCs, a promising next-generation strategy, entered clinical trials. These ADCs can enhance efficacy against heterogeneous tumors and drug resistance. However, they face challenges such as precise, site-specific conjugation and optimizing connector chemistry for two different payloads.

At present, the development directions of ADCs in cancer treatment include: developing novel payloads, improving connectors, identifying reliable predictive biomarkers, and optimizing ADC therapy. Next-generation ADCs will boost efficacy and tumor specificity through double payloads, bispecific targeting, and site-specific conjugation.

In the future, through continuous innovation and research, optimizing ADC design, exploring combination therapies, and strengthening collaboration between clinical and basic research, ADCs are expected to play a bigger role in cancer treatment. They will achieve more efficient and safer tumor therapy and offer more effective options for global cancer patients.

## DECLARATIONS

### Copyright

© The Author(s) 2025.
